# Association of Social Networking Sites Use with Actual and Ideal Body Shapes, and Eating Behaviors in Healthy Young Japanese Women

**DOI:** 10.3390/nu15071589

**Published:** 2023-03-24

**Authors:** Yukina Yumen, Yumi Takayama, Fumiaki Hanzawa, Naoki Sakane, Narumi Nagai

**Affiliations:** 1 Laboratory of Nutritional Physiology, Graduate School of Human Science and Environment, University of Hyogo, Himeji City 670-0092, Hyogo, Japannagai.lab.uh@gmail.com (N.N.); 2 Kyoto College of Nutritional and Medical Sciences, Kyoto 616-8376, Kyoto, Japan; 3 Department of Food Science and Nutrition, School of Human Science and Environment, University of Hyogo, Himeji City 670-0092, Hyogo, Japan; hanzawa@shse.u-hyogo.ac.jp; 4 Division of Preventive Medicine, Clinical Research Institute for Endocrine and Metabolic Disease, National Hospital Organization, Kyoto Medical Center, Kyoto 612-8555, Kyoto, Japan; nsakane@gf6.so-net.ne.jp

**Keywords:** social media, body mass index, body image, nutrition labeling, healthy eating, thinness

## Abstract

Recent reports have associated the use of social networking sites (SNS) with the drive for thinness in young women; however, its influence on their actual body shape and eating behaviors (EB) remains unclear. We aimed to examine the effect of SNS use on body mass index (BMI), body image (BI), and EB in young women. Participants included 196 healthy women (20–29 years) who answered questions about their SNS use, height, weight, BI and EB via a web-based survey. First, the correlation between time spent on SNS and each variable was determined. Participants were then divided into quartiles according to the duration of daily SNS use as long (≥3 h, *n* = 52) and short (<1 h, *n* = 54), and the data were then compared between the groups. Correlation analysis showed that the longer the duration of daily SNS use, the significantly lower the BMI, the use of nutrition labels, and the frequency of consumption of milk and dairy products. The long group had significantly lower BMI and ideal BI than the short group. The results suggest that spending more time on SNS in young women may be associated with thinner actual and ideal body shapes and poorer access to health information and healthy foods.

## 1. Introduction

Underweight (body mass index; BMI < 18.5 kg/m^2^) is found in approximately 10% of adult women worldwide, and female thinness is prevalent not only in countries and regions with inadequate food supplies, but also in those without [1]. One of these countries, Japan, has the highest prevalence of underweight among adult women in the Organization for Economic Cooperation and Development (OECD) and among developed countries [1]. In particular, nearly 20% of young women are underweight [2]. Women in younger generations are more likely to engage in intentional dietary restriction for weight loss, or dieting, due to their greater drive for thinness and desire to achieve a slimmer body shape [3]. The increasing prevalence of thinness among young women has raised concerns about its possible adverse effects not only on women’s own life-course [4], but also on the health of offspring across generations from the Developmental Origins of Health and Disease (DOHaD) perspective [5], highlighting the prevention of thinness among young women as an important public health nutrition issue.

The drive to be thin, which contributes to female thinness, is known to be enhanced by comparison with others [6]. The recent development of social networking sites (SNS), along with a constant stream of attractive platforms for posting images and videos, has dramatically increased the opportunities for comparison with others. The majority of SNS users belong to the younger generations [7]. Several studies have reported associations between SNS use and the drive for thinness in young women [8]. Tiggemann et al. [9,10] showed that the more time female high school students spent on SNS, the higher their drive for thinness. Cohen et al. [11,12] focused on behavioral outcomes associated with SNS use and found that appearance-focused activities on SNS among young adult women promoted thin-ideal internalization and body surveillance, thereby enhancing their drive for thinness. As described above, SNS use has been documented to increase the drive for thinness in adolescent and young adult women, though its influence on their actual and ideal body shapes remains unknown.

Furthermore, given the link between thinness and eating behaviors (EB), the relationship between SNS use and EB also needs to be addressed [13]. Yao et al. [14] reported that among female college students, appearance comparisons on SNS were associated with an increased tendency toward restrained eating. However, the relationships between SNS use and dietary habits and food choices have not been fully elucidated.

This study aimed to clarify the impact of SNS use on actual and ideal body shapes and EB in non-obese young Japanese women.

## 2. Materials and Methods

### 2.1. Participants and Procedure

This cross-sectional study was conducted using a web-based survey between December 2020 and February 2021. Participants were healthy Japanese women over the age of 20 who were recruited through flyers, social media, and emails promoting the study. Exclusion criteria were participants who (1) were pregnant and breastfeeding, (2) had any of the following weight-related medical conditions within the past 5 years: cancer, eating disorders, diabetes mellitus, or endocrine disorders. Participation was voluntary. The study was approved by the Research Committee of the School of Human Science and Environment, University of Hyogo (No.192, 7 December 2018).

Seven hundred women completed questions on demographics, body size, nutrition, and lifestyle (all required questions). However, only the objectified body consciousness questionnaires were non-required questions, resulting in missing values for these questions.

Age and employment status were considered to influence the duration of SNS use, so participants older than 30 years (*n* = 342) and with employment status (*n* = 155) were excluded from the analyses. In addition, seven women reported not having an SNS account (e.g., Facebook, Instagram, Twitter, or TikTok) and were excluded from the analyses, resulting in a final dataset of 196 women between the ages of 20 and 29 years (M = 21.1, SD = 1.8) with a mean reported BMI of 20.3 (SD = 2.3). The prevalence of being underweight (BMI < 18.5 kg/m^2^) and overweight (BMI ≥ 25 kg/m^2^) among all participants was 17.9% and 1.5%, respectively.

### 2.2. Measures

#### 2.2.1. SNS Use

A questionnaire was developed based on a previous report [11]. Participants indicated the number of times they accessed/checked their accounts daily on a 7-point scale: hardly ever, 1 or 2 times, 3–5 times, 5–10 times, 11–15 times, 15–20 times, more times than I can count, and the average amount of time they spent on SNS a day on a 12-point scale: 0–15 min, 15–30 min, 1–2 h, 2–3 h, 3–4 h, 4–5 h, 5–6 h, 6–7 h, 7–8 h, 8–9 h, 9–10 h, 10 or more hours, each scored at the median. Participants also indicated how often they looked at other people’s photos of themselves on a 5-point scale: almost never, rarely, sometimes, often, almost every time I log on. Participants were asked how many selfies they took and posted per day.

#### 2.2.2. Anthropometric Assessments

Participants reported their age, sex, height, and weight. BMI (kg/m^2^) was calculated from self-reported the height and weight data.

#### 2.2.3. Body Image

Body image was assessed using the Japanese version of the Body Image Scale, which has 10 silhouettes ranging from thin (score 1) to obese (score 10). This scale was developed and its reliability and validity were assessed in the Japanese adult population [15]. Regarding reliability, good test–retest reliability was found for both men (*n* = 35, *ρ* = 0.90, *p* < 0.01) and women (*n* = 257, *ρ* = 0.90, *p* < 0.01). In terms of validity, there was a significant positive correlation between the body image score and BMI in both men (*n* = 335, *ρ* = 0.82, *p* < 0.001) and women (*n* = 444, *ρ* = 0.81, *p* < 0.001). The area under the curve (AUC) calculated by sensitivity and specificity was > 0.9 for thinness (BMI < 18.5 kg/m^2^) and obesity (BMI ≥ 25 g/m^2^) in both genders. Body image scores are reliable indicators of thinness or obesity. 

Participants were asked to identify the silhouette that best represented their current body image (CBI) and ideal body image (IBI). The difference between CBI and IBI was calculated as body dissatisfaction discrepancy (CBI−IBI). To assess body perception, participants reported their own body shape on a 5-point scale: thin, slightly thin, normal, slightly overweight, or overweight. Body image distortion was scored based on participants’ body perception and actual body shape: (1) underestimation: normal body shape perceived as thin or slightly thin, and overweight perceived as thin, slightly thin, or normal; (2) overestimation: underweight perceived as normal, slightly overweight, or overweight, and normal body shape perceived as slightly overweight or overweight; (3) no distortion: perceived body shape equal to actual body shape.

#### 2.2.4. Objectified Body Consciousness

The Objectified Body Consciousness Scale [16] was used to assess objectified body consciousness tendencies. The Objectified Body Consciousness Scale consists of three subscales of eight items pertaining to body surveillance, body shame, and appearance control. Participants rate their level of agreement with 24 items (e.g., “I often worry about whether the clothes I am wearing make me look good”) on a 5-point scale (1 = strongly disagree, 5 = strongly agree). McKinley and Hyde (1996) [16] reported good construct and discriminant validity in a sample of female undergraduates. In the present study, alpha was 0.75, 0.74, 0.60, and 0.66 for body surveillance, body shame, appearance control, and total score, respectively. The total score for each subscale (8 items) and the total score for all 24 items were calculated and used for scoring. To consider participants who did not want to answer sensitive questionnaires, only the Objectified body consciousness items were open-ended.

#### 2.2.5. Subjective Feelings

Subjective health perceptions were assessed using a self-report questionnaire [17] that asked, “Do you feel that you are in good health?” Participants reported on a 4-point scale (1 = poor, 4 = excellent), with lower scores indicating lower subjective health perceptions.

The four-item Subjective Happiness Scale [18] was used to measure subjective happiness. Participants reported on a 7-point scale (1 = not a very happy person, 7 = a very happy person) with lower scores indicating lower subjective happiness. The scale has shown good internal consistency in a Japanese undergraduate sample [19]; in the present study, the alpha was 0.88. The average score for the four items was calculated and used for scoring.

#### 2.2.6. Eating Behaviors 

Dietary habits, including skipping breakfast, eating dinner within two hours of bedtime, and eating snacks after dinner were assessed using a self-report questionnaire [20]. Participants answered “yes” if they did so on three or more days per week, and “no” if they did so on two or fewer days per week. In addition, use of nutrition labels was assessed using a self-report questionnaire [21] that asked, “Do you usually refer to the nutrition label when you buy food?” Participants responded on a 4-point scale: hardly ever (0), rarely (1), often (2), and every time (3). 

The frequency of food consumption of fruits, fish, milk and dairy products, and alcohol was assessed using a self-report questionnaire; the food items were selected from the healthy lifestyle behaviors that have been shown to be associated with cardiovascular mortality among Japanese people [22]. Participants reported on a 5-point scale: rarely (0), 1–2 days a month (1), 1–2 days a week (2), 3–4 days a week (3), almost every day (4). Each frequency weight was set to 0, 0.05 (1.5/30), 0.214 (1.5/7), 0.5 (3.5/7), and 1.0.

### 2.3. Statistical Analysis

Using all data (*n* = 196), we evaluated the association between the duration of SNS use and body image and eating behaviors using Spearman correlation coefficients. Based on previous reports [23,24], we compared participants in the highest (long: ≥75th percentile; ≥3 h per day) and lowest (short: <25th percentile; <1 h per day) quartiles of duration of SNS use. Comparisons between the two groups (long vs. short) were performed using the Mann–Whitney U or chi-squared test, as appropriate. Cases with missing data were removed from the relevant analysis and Little’s missing completely at random (MCAR) test was used to demonstrate that the data were missing at random [25]. All statistical analyses were performed using the Statistical Package for the Social Sciences (SPSS for Windows™ ver. 28, IBM Inc., Tokyo, Japan). The significance level was set to alpha = 0.05.

## 3. Results

There were no missing values except for four items of the Objectified Body Consciousness. For the missing data, the number of missing data in the long and short groups was 17 (31.5%) and 15 (28.8%), respectively. Little’s MCAR test showed that the missing data were missing completely at random (Chi-square = 0.09; *p* = 0.768). 

### 3.1. SNS Use

Most participants (88.8%, *n* = 174) checked their SNS account at least 3–5 times per day. Approximately half of the participants (51.0%, *n* = 100) reported using SNS for 2 or more hours per day. Two-thirds of the participants (69.9%, *n* = 137) looked at other people’s photos of themselves often or almost every time they logged on. Approximately one in five participants (17.9%, *n* = 35) habitually took selfies, and only 10 participants (5.1%) posted the photos on SNS (both > 0 times per day).

### 3.2. Relationship between SNS Use and Actual and Ideal Body Shapes, and Eating Behaviors 

**Figure 1:**
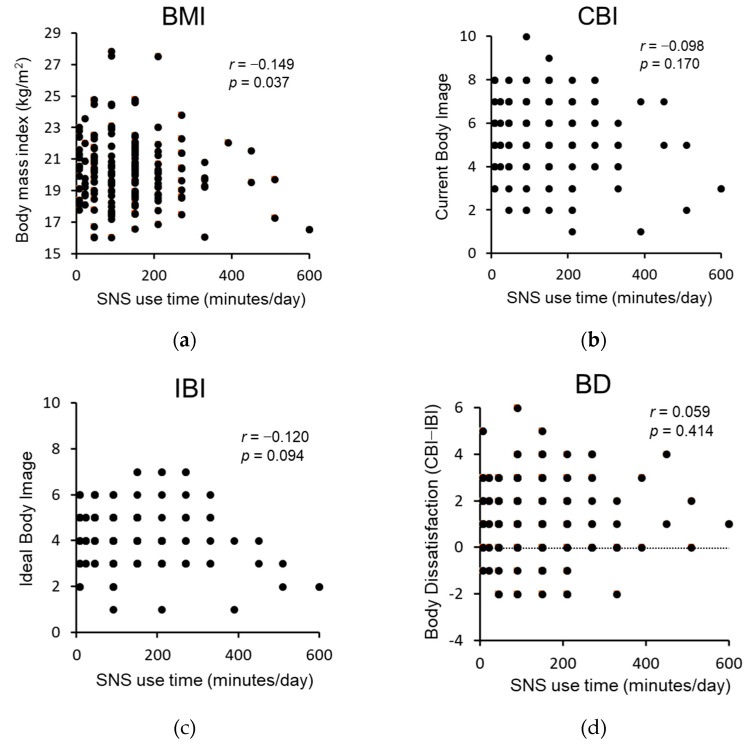
Correlations between the duration of SNS use and (**a**) body mass index, (**b**) current body image, (**c**) ideal body image, and (**d**) body dissatisfaction (all *n* = 196). Spearman’s correlation. Body dissatisfaction was calculated as the difference between current and ideal body image. SNS = social networking sites.

Figure 1 presents the correlation between the duration of SNS use and (a) BMI, (b) CBI, (c) IBI, and (d) body dissatisfaction of all data. BMI was negatively correlated with the duration of SNS use (*r* = −0.149, *p* = 0.037).

Use of nutrition labels (*r* = −0.159, *p* = 0.026) and the frequency of consumption of milk and dairy products (*r* = −0.195, *p* = 0.006) were negatively correlated with the duration of SNS use. Frequency of consumption of fruit, fish, and alcohol was not significantly correlated with the duration of SNS use.

### 3.3. Characteristics in the Long and Short Time Groups of SNS Use 

#### 3.3.1. Daily SNS Use

Table 1 shows the daily SNS use, body shape, body image, Objectified Body Consciousness, and subjective feelings of the long and short groups. Compared to the short group, the long group accessed SNS more frequently (*p* < 0.001), took more selfies (*p* = 0.001), and posted more of them (*p* = 0.001) per day. There were no differences in the frequency of viewing others’ photos of themselves (Table 1).

#### 3.3.2. Body Shape

BMI was lower in the long group than in the short group (*p* = 0.027). Body weight status based on the BMI value did not differ significantly between the two groups.

#### 3.3.3. Body Image

There was no difference in CBI between the two groups (*p* = 0.182). On the other hand, IBI was lower in the long group than in the short group (*p* = 0.026). There were no differences in body dissatisfaction, body perception, and body image distortion between the two groups (Table 1).

#### 3.3.4. Objectified Body Consciousness

The total scores of Objectified Body Consciousness (*p* = 0.032) and body surveillance (*p* = 0.002) were higher in the long group than in the short group. There were no differences in body shame and appearance control between the two groups (Table 1).

#### 3.3.5. Subjective Feelings

The subjective health score was higher in the long group than in the short group (*p* = 0.038). There was no difference in the subjective happiness score between the two groups (Table 1).

#### 3.3.6. Eating Behaviors

Table 2 shows the eating behaviors of the long and short groups. There were significant differences in the frequency of consumption of fruits (*p* = 0.017), milk and dairy products (*p* = 0.045), and alcohol (*p* = 0.043) in the two groups. There was no difference in skipping breakfast, eating dinner within two hours of bedtime, and eating snacks after dinner between the two groups (Table 2).

## 4. Discussion

This study had two main findings: (1) in non-obese young Japanese women, the longer the duration of daily SNS use, the significantly lower the BMI, use of nutrition labels, and frequency of consumption of milk and dairy products; and (2) when comparing subgroups of time spent on SNS, the long group had a lower BMI and IBI than the short group.

### 4.1. SNS Use and Actual and Ideal Body Shapes

A limited number of studies have investigated the association between time spent on SNS and physiques in young women. In contrast with the present finding that the more time young women spent on SNS, the lower their BMI, Wagner et al. [26] reported that there was no correlation between the frequency of posting selfies on SNS and BMI in female college students. This discrepancy is probably due to the methodological difference between the studies, in that Wagner et al. assessed the frequency of posting selfies, one form of SNS activity, whereas we examined the time spent engaging in SNS activity as a whole. This activity mainly consisted of posting and browsing, considering the importance of the total time spent on both components. In 2000, before SNS became an information source, it was suggested that the portrayal of thin models and celebrities on television and in fashion magazines would promote the drive for thinness in young women [27]. As their main source of information is currently shifting from television to SNS [7], our finding that a longer duration of daily SNS use can lead to a thinner physique in young women suggests that further attention needs to be paid to the modes of SNS use in order to reduce thinness among young women.

An interesting finding of the present study was that the long group had a thinner actual and ideal physiques than the short group. Tiggemann et al. [9,10] reported a positive correlation between time spent on SNS and the drive for thinness in adolescent girls. Fardouly et al. [28] found that the more female college students engaged in SNS use, as measured by the mean scores of time spent and frequency, the higher their drive for thinness and body dissatisfaction. The finding from these previous studies that SNS use is associated with increased drive for thinness in young women is in good agreement with our findings that women in the long group who spent more time on SNS had a thinner body size ideal. In the present study, however, no significant difference in body dissatisfaction was noted between the long and short groups. This could be explained by the fact that the long group actually had a thinner physique, with a mean BMI of 19.5 kg/m^2^; therefore, they were less likely to develop greater body dissatisfaction.

### 4.2. SNS Use and Eating Behaviors

The analysis of time spent on SNS and EB revealed that the longer the duration of daily SNS use, the lower the frequency of referring to nutrition labels when purchasing food. The percentage of adult women in Japan [2] who cited SNS as a source of information influencing their diet was highest among those in their 20s (approximately 40%), which, together with our results, suggests that SNS information, rather than reliable information on nutrition labels, is used as a reference for food purchases in young women who spend more time on SNS.

Another interesting new finding of this study was that the longer the duration of daily SNS use, the lower frequency of consumption of milk and dairy products. Previous research on the associations between SNS use and EB found that scores on the Eating Attitudes Test-26 [29] and dieting behavior scores (which measure items such as the frequency of attempts to lose weight) [10] increased with the amount of time spent on SNS. However, to our knowledge, no studies have shown that SNS use affects specific food consumption. In light of this, why were the milk and dairy product intakes lower? In this regard, the study on food selection patterns in Japanese adolescent girls reported that body dissatisfaction was associated with lower frequency of milk consumption [30]. Additionally, a study of Japanese mothers of preschool children found that they were more likely to perceive that “milk and dairy products are high in energy due to their fat content and that consumption of these foods causes weight gain” [31]. In Japan, national surveys have shown a decline in dairy consumption over the past 20 years among non-obese women aged 20–39 years [32]. In reality, however, milk and dairy products are desirable for young women as they are rich sources of nutrients such as calcium and protein. Taken together with the finding on the use of nutrition labels, this finding indicates that the use of SNS as a source of information may have led to limited access to correct information, such as nutrition labels, leading to misconceived avoidance of healthy foods (milk and dairy products). It is plausible that overuse of SNS may be a barrier to access both health information and healthy foods. 

### 4.3. Limitations

The limitations of this study were as follows: (1) A detailed dietary survey was not conducted, and data on participants’ actual food or nutrient intake were not available. (2) SNS use was self-reported, so the possibility of underestimation cannot be excluded [33,34]. In the future, research using objective data logs and screen time applications to assess SNS use is needed [35]. (3) The survey focused primarily on time spent on SNS and not on content viewed and the use of individual application, all of which should be investigated in future studies. (4) The study design was cross-sectional, so cause and effect cannot be inferred. In addition, the possibility of reverse causation cannot be excluded [36]. Future longitudinal or interventional studies are required to confirm our findings. Despite the above limitations, to the best of our knowledge, the present study is the first report to demonstrate the negative impact of prolonged SNS use on both actual and ideal body shapes and healthy EB in non-obese young women.

## 5. Conclusions

The present results suggest that spending more time on SNS may be associated with thinner actual and ideal body shapes and poorer access to health information and healthy foods among young women. In order to prevent thinness and promote healthy EB among young women, it may be necessary to pay attention to the time spent on SNS.

## Figures and Tables

**Table 1:** Daily SNS use, body shape, body image, objectified body consciousness, and subjective feelings by the duration of SNS use.

Variables	Long (*n* = 52)	Short (*n* = 54)	*p*-Value
Daily SNS use ^1^			
Frequency of access ^3^	4.9 ± 1.3	2.1 ± 1.3	**<0.001**
Frequency of viewing others’ photos of themselves ^4^	2.9 ± 1.0	2.8 ± 1.0	0.383
Number of selfies taken	0.4 ± 0.7	0.1 ± 0.4	**0.001**
Number of selfies posted	0.2 ± 0.4	0.0 ± 0.0	**0.001**
Body shape			
Body mass index (kg/m^2^) ^1^	19.6 ± 2.3	20.5 ± 2.0	**0.027**
Body weight status ^2^			0.395
underweight (BMI < 18.5)	11 (21.2)	8 (14.8)	
normal (18.5 ≤ BMI < 25)	40 (76.9)	46 (85.2)	
overweight (25 ≤ BMI)	1 (1.9)	0 (0.0)	
Body Image			
Current body image (CBI) ^1^	5.1 ± 1.8	5.6 ± 1.4	0.182
Ideal body image (IBI) ^1^	4.0 ± 1.4	4.4 ± 1.1	**0.026**
Body dissatisfaction ^1,5^	1.1 ± 1.6	1.2 ± 1.4	0.796
Body perception ^2^			0.194
thin, slightly thin	9 (17.3)	4 (7.4)	
normal	10 (19.2)	21 (38.9)	
slightly overweight, overweight	33 (63.5)	29 (53.7)	
Body image distortion ^2^			0.967
underestimation	1 (1.9)	1 (1.9)	
no distortion	18 (34.6)	20 (37.0)	
overestimation	33 (63.5)	33 (61.1)	
Objectified Body Consciousness ^1,6^			
Body surveillance ^7^	30.4 ± 5.5	26.7 ± 4.9	**0.002**
Body shame ^7^	23.1 ± 6.0	21.8 ± 4.9	0.332
Appearance control ^7^	26.3 ± 4.9	26.9 ± 3.9	0.551
Total	79.8 ± 10.1	75.3 ± 8.4	**0.032**
Subjective feelings ^1^			
Health ^8^	3.1 ± 0.5	2.8 ± 0.7	**0.038**
Happiness ^9^	4.8 ± 1.2	4.7 ± 1.2	0.694

Note. SNS = social networking sites. ^1^ Values represent mean ± SD. *p*-values were analyzed via Mann–Whitney U. ^2^ Values represent *n* (%). *p*-values were analyzed by Chi-square test. ^3^ score range 0–6 (0 = hardly ever, 6 = more times than I can count). ^4^ score range 0–4 (0 = almost never, 4 = nearly every time I log on). ^5^ Body dissatisfaction represents the difference between CBI and IBI (IBI−CBI). ^6^ Long; *n* = 37, Short; *n* = 37 ^7^ score range 8–40 (8 items total score, 1 item score range 1 = strongly disagree, 5 = strongly agree). ^8^ score range 1–4 (1 = poor, 4 = excellent). ^9^ score range 1–7 (1 = not a very happy person, 7 = a very happy person). Bold: significant differences in *p*-Value.

**Table 2:** Eating behaviors by the duration of SNS use.

Variables	Long (*n* = 52)	Short (*n* = 54)	*p*-Value
Skipping breakfast (≥3 days/week)			0.157
Yes	34 (65.4)	42 (77.8)	
No	18 (34.6)	12 (22.2)	
Eating dinner within two hours of bedtime (≥3 days/week)			0.777
Yes	36 (69.2)	36 (66.7)	
No	16 (30.8)	18 (33.3)	
Eating snacks after dinner (≥3 days/week)			0.582
Yes	32 (61.5)	36 (66.7)	
No	20 (38.5)	18 (33.3)	
Nutrition label use			
hardly ever	9 (17.3)	6 (11.1)	0.059
Rarely	10 (19.2)	8 (14.8)	
Often	29 (55.8)	25 (46.3)	
every time	4 (7.7)	15 (27.8)	
Frequency of food consumption			
Fruits			**0.017**
Rarely	11 (21.2)	15 (27.8)	
1–2 days a month	17 (32.7)	9 (16.7)	
1–2 days a week	9 (17.3)	13 (24.1)	
3–4 days a week	12 (23.1)	5 (9.3)	
almost every day	3 (5.8)	12 (22.2)	
Fish			0.357
Rarely	10 (19.2)	6 (11.1)	
1–2 days a month	9 (17.3)	8 (14.8)	
1–2 days a week	27 (51.9)	29 (53.7)	
3–4 days a week	6 (11.5)	8 (14.8)	
almost every day	0 (0.0)	3 (5.6)	
Milk and Dairy products			**0.045**
Rarely	4 (7.7)	3 (5.6)	
1–2 days a month	8 (15.4)	3 (5.6)	
1–2 days a week	20 (38.5)	15 (27.8)	
3–4 days a week	10 (19.2)	8 (14.8)	
almost every day	10 (19.2)	25 (46.3)	
Alcohol			**0.043**
rarely	30 (57.7)	34 (63.0)	
1–2 days a month	11 (21.2)	10 (18.5)	
1–2 days a week	4 (7.7)	10 (18.5)	
3–4 days a week	3 (5.8)	0 (0.0)	
almost every day	4 (7.7)	0 (0.0)	

Note. SNS = social networking sites. Values represent *n* (%). *p*-values were analyzed using Chi-square test. Bold: significant differences in *p*-Value.

## Data Availability

Raw data cannot be shared publicly as it is in a re-identifiable database. These restrictions were placed by the Research Committee of the School of Human Science and Environment, University of Hyogo.
